# Cultivating Reasoning Through Example-Based Or Self-explanation-based Teaching (CREST)?

**DOI:** 10.1007/s40670-025-02612-4

**Published:** 2026-01-19

**Authors:** Chee Chew Yip, Zheng Xian Thng, Francine Peilin Yang, Nicola Yi’an Gan, Andrew Shi-Jie Yap, Jianbin Ding

**Affiliations:** 1 Lee Kong Chian School of Medicine, Nanyang Technological University, Singapore, Singapore; 2 Yong Loo Lin School of Medicine, National University of Singapore, Singapore, Singapore; 3 Department of Ophthalmology & Visual Sciences, Khoo Teck Puat Hospital, Singapore, Singapore; 4 Department of Ophthalmology, Tan Tock Seng Hospital, Singapore, Singapore; 5 Raffles Eye Center, Raffles Specialist Centre, Singapore, Singapore; 6 Ang Mo Kio Polyclinic, National Healthcare Group Polyclinics, Singapore, Singapore; 7 Department of Ophthalmology, National University Hospital, Singapore, Singapore

**Keywords:** Cognitive load theory, Example-Based teaching, Self-Explanation

## Abstract

**Background:**

Example-Based Teaching (EBT), grounded in Cognitive Load Theory, uses modeling to demonstrate expert diagnostic reasoning. Self-Explanation (SE) requires learners to rationalize clinical findings independently. We hypothesized EBT would be superior to SE for teaching clinical reasoning to novices by optimizing cognitive load.

**Methods:**

Twenty-seven second-year medical students without ophthalmology knowledge were randomized to EBT or SE groups. After learning eye fundamentals, participants learned retinal diseases via their assigned method. Clinical reasoning was tested on days 10 and 40 using different case scenarios, graded by a masked assessor. Cognitive load was assessed using a validated questionnaire.

**Results:**

At day 10, the EBT group (*n* = 15) showed significantly higher clinical reasoning scores (median 54.63 vs. 48.15, *p* = 0.047) and germane cognitive load (76.17 vs. 71.25, *p* = 0.016) compared to SE (*n* = 12). No differences emerged at day 40. The questionnaire demonstrated good validity (CVI > 0.9) and reliability (ω > 0.8).

**Discussion:**

EBT was more effective than SE for early clinical reasoning acquisition in novices. The higher germane load with EBT suggests better schema formation, supporting its value in foundational medical education. Larger studies are needed to confirm these findings.

## Introduction

Many diseases often present with overlapping clinical features, making accurate diagnosis challenging even for experienced ophthalmologists. Developing robust clinical reasoning (CR) skills is therefore crucial to reduce diagnostic errors and improve patient outcomes [1].CR is a fundamental competence in medical education, involving data gathering, hypothesis generation, and analytical decision-making [2, 3]. Despite its importance, teaching CR effectively remains complex, as novices often depend on superficial pattern recognition and lack integrated pathophysiological understanding [4, 5].

Teaching CR to novices requires methods that build foundational schema while managing cognitive load moving beyond established approaches like case-based learning, technology-enhanced learning, and simulation [6–10]. This study’s comparison of Self-Explanation (SE) and Example-Based Teaching (EBT), both theory-driven instructional strategies, therefore, offers critical, transferable insights for instructional design and faculty development across clinical disciplines, highlighting how to best structure foundational training to scaffold reasoning skills for novices.

### Self-Explanation

SE is a learning strategy where learners verbalise their reasoning process by explaining, often aloud, the causal and pathophysiological mechanisms underlying clinical findings [11–13]. This process promotes meaningful learning through mental reorganisation and integration of new information into existing biomedical knowledge, fostering the development of coherent mental models of disease [14, 15].

Biomedical knowledge is crucial in SE. It enables novices to understand and diagnose diseases using pathophysiological mechanisms [11, 16]. Diagnostic expertise is then acquired through repeated applications of biomedical knowledge to solve clinical problems [14]. This learning process reorganises knowledge into compact high-level elements (knowledge encapsulation), culminating in the formation of illness scripts [17]. As delineated by the ‘Illness Script Theory’, these patient-oriented scripts encompass the enabling conditions, pathophysiological processes and consequences of diseases for CR [3].

Empirical and meta-analytic evidence demonstrates SE’s effectiveness across domains. Bisra et al., reported moderate-to-large learning gains, particularly when learners were prompted to elaborate causal relationships [18]. Similarly, Chi et al., showed that SE helps reconcile new information with prior knowledge, correcting misconceptions and refining cognitive schemas [19]. In medical education, SE has been shown to improve diagnostic accuracy and promote the integration of basic and clinical sciences [16, 20].

Thus, SE facilitates CR by bridging pathophysiological mechanisms (e.g., ischemia, inflammation) and clinical manifestations (e.g., neovascularisation, retinal swelling), promoting conceptual coherence and flexible diagnostic reasoning across clinical contexts.

### Example-Based Teaching

EBT involves presenting worked examples that illustrate expert reasoning in diagnosing and managing diseases to enhance diagnostic knowledge and retention [21, 22]. Learners observe the sequence of diagnostic steps, differentiating between similar presentations, understand how diagnostic decisions are structured and justified and learn through imitation [23–26]. Grounded in Cognitive Load Theory (CLT), EBT is more efficient than problem-solving approaches because it directs cognitive resources toward learning and schema formation rather than inefficient trial-and-error reasoning [23, 27].

EBT may be ideal for teaching CR as it provides illness scripts that may reduce intrinsic cognitive load [28]. Furthermore, studying a variety of clinical examples promotes deep conceptual understanding and more illness scripts formation [2, 23]. This rich collection of scripts is required for diagnostic reasoning [3].

### Theoretical & Practical Considerations

SE is a constructive self-learning method whereby illness scripts are developed after going through a staged process of causal network (of concepts) formation and knowledge encapsulation [5, 29]. In contrast, EBT presents experts’ illness scripts directly to the learner to reduce the cognitive load for learning [7].

Guided by the Cognitive Load Theory, we hypothesise that EBT offers a more pragmatic and efficacious approach to teach CR [28]. By explicitly modelling the expert’s diagnostic process, EBT reduces the cognitive burden on learners who might otherwise struggle to construct diagnostic pathways with limited biomedical knowledge. Germane cognitive load, defined as the mental effort devoted to constructing and automating schemas in long-term memory, plays a central role in effective learning [30]. Instructional strategies that enhance germane load, without increasing extraneous or intrinsic load, are more likely to facilitate meaningful schema acquisition. EBT supports this process by directing cognitive resources toward schema construction through expert exemplars, thereby offering a more efficient pathway to develop CR.

We believe that with experts explicating their schemas through EBT, learners can devote more mental resources towards schema formation, which may translate into greater CR gain and more optimal cognitive load. As such, this pilot study was designed to produce preliminary evidence, evaluate the feasibility, and set grounds for future trials to compare EBT and SE in teaching retinal diseases.

### Hypotheses

1. Compared against SE, learning with EBT results in better CR in medical students without prior Ophthalmology related knowledge.
2. EBT poses a more optimal cognitive load than SE during learning.

## Materials and methods

This randomized pilot study was conducted at Lee Kong Chian School of Medicine, Nanyang Technological University, Singapore, from October to December 2022. Ethics approval was obtained from the university’s Institution Review Board, and the study adhered to the tenets set forth in the Declaration of Helsinki.

### Participants

Following the guidance by Eldridge et al., a sample size of 24–50 participants is considered adequate to estimate effect sizes and assess logistical feasibility [31]. Recruitment was opened to the entire second year undergraduate cohort (120 medical students) from the university. Thirty-two provided informed consent for this investigative educational intervention outside the curriculum. These pre-clinical students had no prior knowledge of Ophthalmology because it was not part of their curriculum.

Subjects were assigned to either the SE-group or the EBT-group via manual randomization using a random number generator. Group assignments were disclosed to subjects at the start of the study, while assessors were blinded to reduce observer bias.

### Materials

All instructional materials were developed by four consultant ophthalmologists with over eight years of experience as clinician educators trained in clinical instruction and assessment. The materials were created using Microsoft PowerPoint (Microsoft Corporation, Redmond, Washington). The teaching sessions in both groups were matched for duration and cognitive content to ensure instructional equivalence, isolating the pedagogical mechanism as the variable of interest.

#### Face-To-Face Introductory Session

The was a one-hour, joint introductory lecture to establish foundational knowledge. This session covered basic ocular anatomy and the underlying pathophysiology of retinal diseases, including mechanisms of ischemia, inflammation, and degeneration. This foundational knowledge ensured all participants had comparable baseline understanding prior to the intervention.

#### Group-Specific Teaching Intervention

This was a 90-minute session divided into three 30-minute modules, each dedicated to a different retinal disease. The EBT group learned through worked examples where tutors modeled diagnostic reasoning, while the SE group received disease-based lectures and were guided to actively link clinical findings to underlying mechanisms.

##### Example-Based Teaching (EBT) Session

Six worked examples were used to teach the EBT group. Three diseases were covered with two worked examples each: diabetic retinopathy (DR), age-related macular degeneration (AMD), and retinal vein occlusion (RVO). These cases presented typical histories, hallmark clinical signs, and key investigation results.

The tutors led structured case walkthroughs that modelled expert diagnostic reasoning. The tutor verbalised the sequence of diagnostic steps, elucidated key features for differentiating look-alike diseases, and explained the clinical rationale using biomedical principles. This aligns with the instructional principles of worked examples, where expert reasoning pathways are made visible to reduce cognitive load and promote schema acquisition [23]. Unlike traditional lectures, these sessions emphasised the modelling of diagnostic thought processes, consistent with the modelling-example framework shown to foster deep learning [26].

##### Self-Explanation (SE) Teaching Session

Three structured disease-based lectures were used to teach the SE group. Three diseases (DR, AMD, and RVO) were covered with one lecture each. Of note, each disease is covered by a single tutor who teaches both the EBT and SE group for consistency. Each lecture covered disease mechanisms, hallmark clinical features, and diagnostic criteria.

The tutors demonstrated SE by linking biomedical concepts to clinical features, modelling how to generate pathophysiological explanations for signs and symptoms. Students were then prompted to verbalise their own explanations of practice cases using the same framework. This instructional method promotes cognitive integration by encouraging learners to reorganise biomedical knowledge into diagnostic schemas through elaboration and causal reasoning [11, 20]. Unlike the EBT condition, learners in the SE group had to construct diagnostic pathways independently, without being shown complete expert exemplars.

#### Criterion Cases (Practice)

Criterion cases are practice cases with similar diagnosis and presentations to those covered during the teaching sessions. The criterion cases served to consolidate learning and simulate diagnostic practice. Tutor guidance was specifically designed to reinforce each group’s distinct reasoning methodology, not to alter it. Following each teaching session, students practised six criterion cases (two per disease group) under tutor guidance. The EBT group was guided to apply expert-derived reasoning strategies (modelled in the worked examples), while the SE group was guided to perform self-explanation based on biomedical knowledge to arrive at diagnoses. Tackling these criterion cases under tutor guidance helped to consolidate learning and simulate authentic diagnostic reasoning.

### Instruments

#### Cognitive Load Questionnaire

This is a 10-item, previously validated questionnaire that measures three different types of cognitive load (Appendix 1) [32]: Intrinsic Load (Items 1, 2, and 3), Extraneous Load (Items 4, 5, and 6), and Germane Load (Items 7, 8, 9, and 10). Each item can be rated from 1 to 10, proportionate to the level of cognitive load. The questionnaire measures three distinct types of mental effort. A high score on Intrinsic Load indicates high task complexity involving more learning elements and element interactivity, while a high score on Extraneous Load suggests the instruction itself was poorly designed and inefficient. Crucially, a high score on Germane Load reflects higher cognitive processing devoted to learning and schema construction.

The questionnaire was re-validated to ensure its applicability to our study. Content validity was assessed using the Content Validity Index (CVI) [33]. The expert review panel was formed by 10 consultant Ophthalmologists, of which 4 were study investigators (Yip CC, Yang FP, Thng ZX, Gan NY) and 6 were independent external experts. All experts were accredited specialists by the Singapore Medical Council with more than 8 years of experience as core faculty members for undergraduate medical education.

Experts were asked to grade the necessity and appropriateness of each item using a 4-point Likert scale (1 – not relevant, 2 – somewhat relevant, 3 – quite relevant, 4 – highly relevant). CVI was calculated both at the item (I-CVI) and scale (S-CVI) levels using previously published formulas [34]. For S-CVI, we adopted both the average and unanimous agreement method to ensure scale level content validity. The acceptable value was 0.8 for I-CVI and S-CVI (unanimous agreement) and 0.9 for S-CVI (average) [33].

Internal reliability of the questionnaire was assessed using the more robust McDonald’s Omega (ω), given the small sample size that does not satisfy the normality assumption for Cronbach’s alpha statistics [35]. Each type of cognitive load was analysed as separate constructs. Further divisions were made based on the type of teaching method, as well as the disease group assessed. This produced 18 independent ω values to ensure the internal reliability of the questionnaire was repeatable for every subgroup assessed.

#### Short Answer Questions Test (SAQT)

CR was assessed using two SAQTs, each comprised of nine test cases. These cases included a mix of standard cases (identical to criterion cases, which presented typical clinical features of the taught diseases), and variant cases (which required learners to recognise the same diagnosis despite atypical presentations).

Each disease group was represented by six test cases, of which four were standard and two were variant. The SAQT scoring scheme, which evaluated diagnostic accuracy and justification of reasoning, is detailed in Appendix 2.

### Procedures

**Fig. 1:**
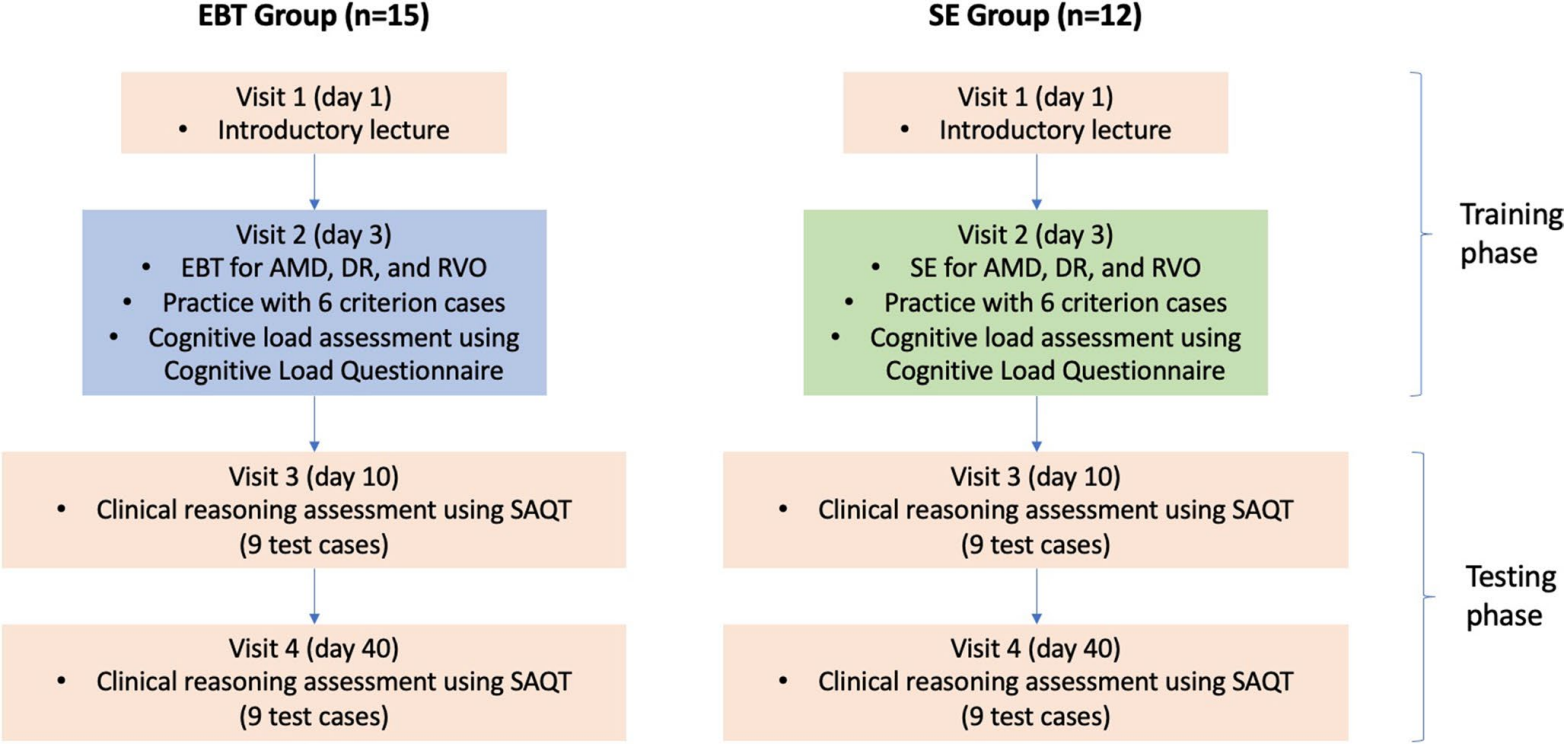
Summary of study procedures for all 4 study visits

The study procedures (Fig. 1**)** were adapted from the study by Chamberland et al. [36]. Our training was spaced out over two visits to reduce cognitive load. We assessed the gain and longitudinal change in CR (SAQT scores) assessed through two testing visits at day-10 and day-40.

#### Training Phase

##### Visit 1 (day-1)

All participants attended a joint, one-hour face-to-face introductory lecture covering basic ocular anatomy and the pathophysiological mechanisms of retinal diseases. This session was designed to establish a common baseline of foundational knowledge across both groups.

##### Visit 2 (day-3)

Participants returned for the main instructional intervention. Teaching was conducted by three consultant ophthalmologists, each responsible for one of the three disease groups (DR, AMD, and RVO). Each group (EBT and SE) underwent three consecutive teaching sessions (30 min per disease group), separated by 10-minute breaks, totalling 90 min of instructional time.

In the EBT group, tutors modelled diagnostic reasoning using worked examples. They walked learners through each case, explicated diagnostic steps, and embedded biomedical knowledge to highlight discriminating features. In the SE group, tutors delivered structured, disease-based lectures and demonstrated self-explanation strategies. Students were then guided to actively link clinical findings to disease mechanisms through verbal reasoning.

Following each teaching session, students completed two practice cases per disease group to consolidate learning. The EBT group applied the expert reasoning demonstrated in the worked examples, while the SE group engaged in guided self-explanation. At the end of Visit 2, participants completed the 10-item Cognitive Load Questionnaire.

#### Testing Phase

##### Visits 3 and 4 (days-10 and 40)

One SAQT was administered each visit to both groups, and 90 min were allocated for each assessment. The SAQT was graded by an assessor who was blinded to the students’ group assignments. The assessor was entirely independent of the instructional team, ensuring grading was based solely on answer correctness and precluding any potential for bias.

### Statistical Analysis

Statistical analysis was performed using IBM^®^ SPSS^®^ Statistics (version 28, SPSS inc, Chicago, Illinois). Due to the small sample size and the ordinal nature of the data, which was not assumed to be normally distributed, non-parametric tests were employed. Data are presented as median with inter-quartile range. The Mann-Whitney U test was used for inter-group comparisons (e.g., EBT vs. SE on SAQT 1 and cognitive load scores), while the Wilcoxon Signed-Rank test was used for intra-group comparisons of repeated measures (e.g., SAQT 1 vs. SAQT 2 within each group). A p-value of < 0.05 was defined as statistically significant.

## Results

Three subjects from the SE group dropped out of the study after the visit 1 and one subject each from each group were excluded due to incomplete test responses. A total of 12 subjects from the SE group and 15 subjects from the EBT group were included for the final data analysis, giving a total sample size of 27.

### Clinical Reasoning Test Results

The SAQT 1 (day-10) score was significantly higher in the EBT group than the SE group (59.00 [19.00] versus 52.00 [17.00], *p* < 0.05). There was no significant difference between EBT and SE group for SAQT 2 (day-40) (58.00 [19.00] versus 52.00 [17.25], *p* = 0.49) (Table 1).

**Table 1:** SAQT score inter-group comparison

	SAQT 1		SAQT 2	
	EBT	SE	EBT	SE
Median	59.00	52.00	58.00	52.00
Inter-quartile range	19.00	17.00	19.00	17.25
Mann-Whitney U test p-value	< 0.05		0.49	

There was no significant difference between SAQT 1 and SAQT 2 scores within either group (Table 2): the EBT group (59.00 [19.00] vs. 58.00 [19.00], *p* = 0.10) and the SE group (52.00 [17.00] vs. 52.00 [17.25], *p* = 0.30).

**Table 2:** SAQT score intra-group comparison

	EBT		SE	
	SAQT 1	SAQT 2	SAQT 1	SAQT 2
Median	59.00	58.00	52.00	52.00
Inter-quartile range	19.00	19.00	17.00	17.25
Wilcoxon-Signed Rank test p-value	0.10		0.30	

### Cognitive Load Questionnaire Validation

All items in this questionnaire achieved an I-CVI of at least 0.9, with an overall S-CVI of 0.98 using the average method and 0.80 using the unanimous agreement method (Table 3). In terms of internal reliability, ω was above 0.80 for all subgroups and above 0.90 for 16 out of 18 subgroups tested (Table 4).

**Table 3:** Content validity index grading outcomes

	Study Team				External Experts						CVI	
	PI	CI-1	CI-2	CI-3	1	2	3	4	5	6	I-CVI	S-CVI
Q1	4	4	3	4	4	4	4	4	4	4	1	Average: 0.98 Unanimous agreement: 0.80
Q2	4	4	4	4	3	4	4	3	4	4	1	
Q3	4	4	4	4	4	4	4	4	4	4	1	
Q4	4	4	4	4	4	4	4	2	3	4	0.9	
Q5	4	4	4	4	4	4	4	4	3	4	1	
Q6	4	4	4	4	4	4	4	2	3	3	0.9	
Q7	4	4	4	4	4	4	4	4	3	4	1	
Q8	4	4	4	4	4	4	4	4	3	3	1	
Q9	4	4	4	4	4	4	4	4	3	4	0.9	
Q10	4	4	4	4	4	4	4	4	3	4	1

**Table 4:** Internal reliability assessment of the cognitive load questionnaire using the mcdonald’s ω statistic

Group	AMD			DR			RVO		
	Intrinsic	Extraneous	Germane	Intrinsic	Extraneous	Germane	Intrinsic	Extraneous	Germane
**Control**	0.975	0.891	0.964	0.966	0.916	0.974	0.961	0.923	0.961
**Intervention**	0.924	0.949	0.874	0.948	0.944	0.939	0.962	0.934	0.934

### Cognitive Load Analysis

The EBT group reported significantly higher germane cognitive load compared to the SE group (95.00 [16.00] vs. 85.50 [12.75], *p* < 0.05), suggesting greater cognitive engagement in schema construction and learning processes. However, no significant differences were found between the two groups in intrinsic load (39.00 [19.00] vs. 36.50 [18.50], *p* = 0.52) or extraneous load (10.00 [15.00] vs. 16.00 [21.50], *p* = 0.55) (Table 5). Additionally, no statistically significant correlations were identified between any of the cognitive load components and SAQT performance.

**Table 5:** Cognitive load analysis summary

	Intrinsic		Extraneous		Germane	
	EBT	SE	EBT	SE	EBT	SE
Median	39.00	36.50	10.00	16.00	95.00	85.50
Inter-quartile range	19.00	18.50	15.00	21.50	16.00	12.75
Mann-Whitney U test p-value	0.52		0.55		< 0.05	

## Discussion

This pilot study compared EBT and SE for teaching ophthalmology to pre-clinical students, finding that EBT led to superior short-term clinical reasoning scores and higher germane cognitive load. However, this advantage was not sustained at the 40-day follow-up. These results demonstrate feasibility and indicate EBT’s promise for improving initial learning, warranting further investigation in a larger cohort.

Given the vastly different educational context from the questionnaire’s original validation on Dutch PhD students in social and health sciences learning mathematics, re-validation was crucial [32]. This first re-validation in undergraduate medical education demonstrated excellent content validity at both item and scale levels with satisfactory CVI scores, supported by desirable S-CVI outcomes from both calculation methods [37]. The questionnaire also showed excellent internal reliability across all cognitive load domains and study groups, with satisfactory ω values, justifying its application in our setting [38].

The significantly better SAQT performance in the EBT group indicates superior clinical reasoning, supporting our hypothesis that EBT is more efficacious than SE for medical novices. This early advantage at 10 days post-teaching may reflect EBT’s direct approach in rapidly transmitting clinical reasoning knowledge through explicit demonstration of expert thought processes, essentially ‘teaching students how to think’. In contrast, SE requires learners to rely on their limited biomedical knowledge to diagnose diseases and create clinical linkages through repetitive practices, which may be less effective for novices who are more likely to construct mental models with false concepts that perpetuate during knowledge encapsulation.

This advantage is further explained by the CLT. The EBT group reported significantly higher germane cognitive load compared to the SE group, suggesting greater cognitive engagement in schema assimilation, and learning processes. This indicates their cognitive resources were more effectively channelled into building and refining mental models, due to the effective instructional design of the worked examples [27, 30]. This efficient schema assimilation likely underpinned their superior early diagnostic performance. The finding that well-constructed worked examples improve learning outcomes by optimizing cognitive load is well-supported in established literature [8, 23, 26].

In this study, the median extraneous load was low for both groups: 10 for EBT and 16 for SE. This may be due to the deliberate design of learning materials that minimised unnecessary text and visuals, as extraneous load is influenced by information presentation and instructional format [30]. Although the difference between groups was not statistically significant, we hypothesise that EBT may indeed impose a lower extraneous load, and our study was not sufficiently ‘powered’ to detect. Prior studies support this interpretation, as students engaging in SE during a virtual reality biology class reported higher extraneous load [39], while worked examples used in psychotherapy pedagogy and physiotherapy CR training reduced extraneous load, enhanced learning efficiency, and supported skill acquisition [40, 41]. These findings suggest that EBT may confer a genuine advantage in lowering extraneous load compared with SE. This hypothesis warrants validation in future larger-scale studies, which may use our results as pilot data.

There was no significant difference in intrinsic cognitive load between learners taught using EBT and SE. This finding was expected, as intrinsic load is determined by the number and interactivity of task elements and the learner’s expertise [30, 42]. In our study, both groups received identical educational content and completed the same six criterion practice cases. Participants were also drawn from a single cohort without prior Ophthalmology experience and were randomly allocated to either EBT or SE. This reduced selection bias, thereby ensuring comparable baseline knowledge. Of note, the median intrinsic load scores for both groups were below 40 (out of 100), suggesting manageable task complexity that was unlikely to impede learning. This is likely an outcome of our deliberate focus on three closely related retinal diseases, which limited cognitive demand and was appropriate for novice learners. We hypothesize that EBT, given its established role in managing cognitive load, may be particularly beneficial for teaching more complex tasks with higher intrinsic load to advanced trainees. Future studies may therefore compare EBT and SE in training Ophthalmology residents to diagnose and manage more conceptually demanding conditions such as retinal dystrophies or neuro-ophthalmic disorders.

We did not find any statistically significant associations between cognitive loads and SAQT performance, this could be explained by our study’s small sample size and the fact that sample size calculation was not performed. Of note, we also found no significant difference in SAQT performance at 40 days post-learning. This could be explained by the lack of practice to consolidate knowledge between tests and is not unexpected. Future studies may consider incorporating practice sessions between the assessments using EBT and SE based learning materials, this may better compare their effect on teaching CR in a setting that resembles actual learner behaviour.

The study findings highlight a critical principle for teaching complex reasoning across disciplines: for novices, explicitly modelling expert thought processes is more effective than discovery-based learning. The success of EBT demonstrates that instructional design must optimize cognitive load by providing structured worked examples, which efficiently build accurate mental models. This necessitates a shift in faculty development from content delivery training to coaching educators in how to design learning experiences that systematically scaffold expert cognitive processes.

Our study has three main strengths. Firstly, the selection of medical undergraduates without prior eye training removed pre-existing knowledge as a confounder. Secondly, our instrument selection (a previously validated cognitive load questionnaire) and re-validation ensured the applicability of instrument in our education context, facilitating reliable measurement of cognitive load. Notably, a greater number of independent experts without conflicts of interest participated in the CVI grading, which helped reduced bias. Thirdly, SAQT provides a deep assessment of clinical reasoning, problem-solving, and knowledge recall. It also reduces guessing and allows evaluation of a candidate’s thought process, as compared to multiple choice questions.

Our study has two main limitations. Firstly, the small sample size, further impacted by attrition, aligns with the feasibility objectives of this pilot study. While this limits the statistical power and generalizability of the findings, it successfully informs a future definitive trial. Based on the effect sizes observed, a formal sample size calculation is now possible to ensure adequate power for robust hypothesis testing in subsequent research. Secondly, the study focused solely on quantitative cognitive load measures, which, though validated, may not fully capture the complex reasoning processes involved in clinical diagnosis. The exclusive focus on retinal disorders also limits applicability across other medical domains with differing cognitive demands. A mixed-methods approach, incorporating qualitative data such as learner reflections or think-aloud protocols, could provide deeper insight into how instructional strategies like EBT and SE influence the development of clinical reasoning.

## Conclusion

In summary, this study found EBT to be more efficacious than SE in teaching CR to medical novices. This could be explained by a more optimal cognitive load, assessed using a successfully re-validated questionnaire, and mainly contributed by germane load increments in EBT-taught learners.

## Data Availability

Data is stored as hardcopy documents within the Principal Investigator’s (Professor Chee Chew Yip) institution and may be retrieved for review upon reasonable request.

## Appendix 1

**A ten-item questionnaire to measure different cognitive loads: Intrinsic Load (Items 1, 2, and 3), Extraneous Load (Items 4, 5, and 6), and Germane Load (Items 7, 8, 9, and 10).**

The following questions refer to the e-learning and 2 face-to-face teachings sessions that you have completed.

Please rate each of the questions with a score ranging from 0 to 10. A score of “0” means not the case and “10” means completely the case.

1. The topic/topics covered in the activity was/were very complex.
2. The activity covered diagnostic steps that I perceived as very complex.
3. The activity covered concepts and definitions that I perceived as very complex.
4. The instructions and/or explanations during the activity were very unclear.
5. The instructions and/or explanations were, in terms of learning, very ineffective.
6. The instructions and/or explanations were full of unclear language.
7. The activity really enhanced my understanding of the topic(s) covered.
8. The activity really enhanced my knowledge and understanding of disease diagnosis.
9. The activity really enhanced my understanding of the diagnostic steps covered.
10. The activity really enhanced my understanding of concepts and definitions.

**Note: **

**Questions 8 and 9** have been modified for use in medical case diagnosis. The original terms “statistics” (question 8) and “formula” (questions 2 & 9) from the original questionnaire have been changed to “disease diagnosis” and “diagnostic steps” respectively.

## Appendix 2

**Table:**

	Answer
Diagnosis (2)	Left non-proliferative diabetic retinopathy
List one complication, if any (1)	Diabetic macular oedema
Two key examination findings from the colour fundus photo (2)	Dot haemorrhage or blot haemorrhage Hard exudate Flame shaped haemorrhage
One main point in the history to support your diagnosis, or refute an important differential (1)	Supporting History of diabetes Refuting Absence of sudden visual loss
One additional history point to obtain (1) and why (1)	Duration of diabetes Control of diabetes
One main diagnostic feature in the investigation result shown (excluding fundus photo) (1)	Cystoid macular oedema Absence of retinal pigment epithelium
One more important investigation to be performed (1) and why (1)	Fundus fluorescein angiography - To evaluate for neovascularization, capillary fallout, macular oedema Amsler chart - To demonstrate metamorphopsia
One main differential diagnosis (1)	Age-related macular degeneration

## Declarations

### Deta Deposition

Offline hardcopy documents within Khoo Teck Puat Hospital, Singapore.

## Abbreviations

AMD: Age-related Macular Degeneration
CR: Clinical Reasoning
CLT: Cognitive Load Theory
CVI: Content Validity Index
DR: Diabetic Retinopathy
EBT: Example-Based Teaching
I-CVI: Item-level Content Validity Index
RVO: Retinal Vein Occlusion
SAQT: Short Answer Question Test
S-CVI: Scale-level Content Validity Index
SE: Self-Explanation
